# Cryptococcal Meningoencephalitis with Possible Clinical-Manometric Intracranial Pressure Dissociation in a Pediatric Patient

**DOI:** 10.3390/pediatric18050125

**Published:** 2026-09-24

**Authors:** Fabrício Silva Pessoa, Valéria Maciel Costa, Izabel Athayde da Silva Cruz Salgado

**Affiliations:** Pediatric Infectious Diseases Service, University Hospital of the Federal University of Maranhão (HU-UFMA), Sao Luis 65070-020, Brazil; valeriamacielcosta@gmail.com (V.M.C.); izabel.athayde@yahoo.com.br (I.A.d.S.C.S.)

**Keywords:** cryptococcal meningoencephalitis, intracranial hypertension, pediatric, *Cryptococcus gattii*, intracranial pressure monitoring, immune reconstitution inflammatory syndrome

## Abstract

A 12-year-old previously healthy girl without an identified immunodeficiency presented with a 25-day history of headache, vomiting, and severe asthenia. Cerebrospinal fluid (CSF) analysis confirmed *Cryptococcus gattii* meningoencephalitis. Despite consistently normal intracranial pressure (ICP) readings on serial lumbar punctures (all below 20 cm H_2_O), the patient developed clinical signs suggestive of raised intracranial pressure, progressing to decreased consciousness requiring ICU admission and orotracheal intubation. Brain MRI demonstrated communicating hydrocephalus, leptomeningeal enhancement, and perineural contrast enhancement of the optic nerve sheath and of cranial nerves VII and VIII. Management included an external lumbar subarachnoid drain for CSF drainage and antifungal induction with liposomal Amphotericin B combined with Flucytosine, in accordance with the 2024 ECMM/ISHAM/ASM Global Guideline for non-HIV CNS cryptococcosis. Notwithstanding treatment, the patient developed irreversible amaurosis and bilateral sensorineural hearing loss attributed to perineural leptomeningeal dissemination, confirmed on MRI. This case illustrates a possible clinical-manometric ICP dissociation in pediatric cryptococcal meningoencephalitis—a phenomenon that may reflect adhesive arachnoiditis with spinal CSF compartmentalization rather than true ICP elevation—and underscores the importance of multimodal assessment beyond intermittent manometry in this setting.

## 1. Introduction

Cryptococcal meningoencephalitis is an important cause of mortality and neurological morbidity in children, caused predominantly by members of the *Cryptococcus* species complex. *Cryptococcus neoformans* is the leading species in HIV-associated pediatric cases, particularly in sub-Saharan Africa, whereas *C. gattii*, *C. laurentii*, *C. albidus* and *C. neoformans* var. *grubii* have all been described in immunocompetent pediatric hosts [1,2,3,4,5,6,7,8,9]. Among non-HIV-infected patients, neurological sequelae affect up to 73% of survivors at 12-month follow-up [1]. The most common pediatric neurological complications include visual impairment—highly associated with raised intracranial pressure—learning difficulties, recurrent seizures, and sensorineural hearing loss [3,10]. Elevated ICP is recognized as one of the strongest independent predictors of poor outcome, yet its reliable assessment by intermittent lumbar manometry remains a major clinical challenge, as spot measurements capture only a snapshot of a continuously fluctuating waveform [11]. Herein, we report the case of a 12-year-old girl without an identified immunodeficiency with *C. gattii* meningoencephalitis who exhibited clinical signs suggestive of refractory ICH with a possible clinical-manometric dissociation, and discuss the diagnostic, monitoring, and therapeutic implications of this underrecognized phenomenon.

## 2. Case Report

A 12-year-old female patient was admitted with headache, vomiting, and severe asthenia for 25 days. She was afebrile on admission and had previously received empirical Ceftriaxone for suspected bacterial meningitis at the referring facility. Laboratory workup revealed: hemoglobin 12 g/dL; leukocytes 13,751/mm^3^ (lymphocytes 1004; neutrophils 12,252; monocytes 421; eosinophils 62; basophils 12 cells/mm^3^); platelets 331,000/mm^3^; C-reactive protein 0.8 mg/dL; and erythrocyte sedimentation rate 26 mm/h. CSF analysis on admission revealed 464 leukocytes/μL, 10 erythrocytes/μL, glucose 31 mg/dL, and total protein 62.8 mg/dL. All lumbar punctures were performed in the lateral decubitus position with legs extended, with the manometer zeroed at the level of the lumbar puncture site. The opening pressure on admission was 16 cm H_2_O; all subsequent punctures during hospitalization yielded opening pressures below 20 cm H_2_O. The first two measurements were obtained while the patient was awake and spontaneously breathing; subsequent measurements after ICU admission were obtained with the patient under sedation and mechanically ventilated (midazolam and fentanyl infusion), with mechanical ventilation temporarily paused and the endotracheal tube occluded during measurement to minimize positive-pressure artifacts. Each measurement was performed at least six hours after the preceding CSF drainage procedure.

Despite consistently normal manometric values, the patient deteriorated markedly within four days, developing clinical signs suggestive of raised ICP refractory to daily CSF-relief lumbar punctures: progressive headache, papilledema on fundoscopy, and rapid deterioration of consciousness (Glasgow Coma Scale score falling from 14 to 10/15). It should be noted that papilledema in the context of active cryptococcal meningoencephalitis may reflect either raised ICP or direct infiltration of the optic nerve sheath, and the relative contribution of each mechanism cannot be determined in the absence of direct ICP measurement. She required ICU admission and orotracheal intubation. India ink staining of CSF revealed encapsulated yeasts consistent with *Cryptococcus* spp., and culture subsequently identified *Cryptococcus gattii* as the causative agent. Species identification was performed by MALDI-TOF mass spectrometry (Vitek MS system, bioMérieux), confirmed by biochemical profile (API 20C AUX). Antifungal susceptibility testing was not performed routinely at our institution. Serum CrAg (lateral flow assay) was not available at admission; a serum CrAg titre of 1:2048 was obtained on day 7, consistent with high fungal burden. Repeat CSF cultures were obtained at weeks 2, 4, and 6; positivity persisted through week 4 and converted to negative at week 6. Antifungal induction was initiated with conventional Amphotericin B deoxycholate and Fluconazole, reflecting local formulary constraints, and subsequently escalated to liposomal Amphotericin B (3–4 mg/kg/day) combined with Flucytosine (100 mg/kg/day in four divided doses), in accordance with the 2024 ECMM/ISHAM/ASM Global Guideline for non-HIV CNS cryptococcosis [12]. Given the failure of serial lumbar punctures, an external lumbar subarachnoid drain (placed using an epidural catheter kit at the L3–L4 interspace) was inserted on day four and maintained for 21 days, averaging 10–15 mL/hour. Opening pressures recorded after drain placement (days 5 onward) were obtained by temporarily clamping the drain for a minimum of six hours prior to measurement, then performing a separate lumbar puncture needle insertion at an adjacent interspace (L2–L3) to avoid measuring through the drain itself. These conditions are acknowledged as a limitation, as the clamped state may not fully reflect the patient’s untreated intracranial pressure dynamics. Drain replacement was required following drain-associated meningitis caused by *Pseudomonas aeruginosa* (diagnosed on CSF culture obtained through the drain), treated with Meropenem. Despite interventions, the patient developed progressive visual and auditory loss, bilateral papilledema, and brain MRI with gadolinium demonstrated: (i) dilation of the lateral ventricles consistent with communicating hydrocephalus; (ii) leptomeningeal enhancement; (iii) contrast enhancement of the right optic nerve sheath—a finding consistent with perineural inflammation or infiltration rather than a specific marker of raised ICP; (iv) enhancement of cranial nerves VII and VIII bilaterally; and (v) dilated perivascular spaces consistent with gelatinous pseudocyst formation. No cryptococcomas or parenchymal signal changes were identified.

After six weeks of induction therapy, CSF cultures yielded no growth, though direct India ink examination remained positive. The patient was considered without an identified immunodeficiency based on: HIV Ag/Ab negative; no prior history of recurrent infections, malignancy, transplant, or immunosuppressants; CD4^+^ 920 cells/μL; normal serum immunoglobulins; normal lymphocyte subsets; anti-GM-CSF autoantibodies negative. Suspected paradoxical IRIS was diagnosed based on: (i) paradoxical clinical worsening after initiation of effective antifungal therapy; (ii) improving microbiological markers (CSF culture negative at week 6); and (iii) an intensified inflammatory CSF profile on repeat analysis. Methylprednisolone pulse therapy (30 mg/kg/day × 3 days) and intravenous human immunoglobulin (2 g/kg) were administered per guideline recommendations for CNS IRIS [13], without clinically meaningful response. The absence of response, combined with MRI-confirmed progressive perineural enhancement, suggested that structural leptomeningeal injury—rather than immune dysregulation alone—was the predominant driver. Ventriculoperitoneal shunting was discussed but not performed given concurrent active CNS infection and absence of progressive ventriculomegaly. Maintenance antifungal therapy was initiated with oral fluconazole, later replaced by liposomal Amphotericin B (3 mg/kg/day, three times weekly) due to grade 3 hepatotoxicity. At eight-month follow-up, the patient had irreversible bilateral amaurosis (no light perception bilaterally) and bilateral severe-to-profound sensorineural hearing loss confirmed on pure-tone audiometry. She was referred for cochlear implant evaluation, low-vision rehabilitation, and neurodevelopmental assessment. The clinical course is summarized in Table 1, and a schematic overview of the case is provided in Figure 1.

This study was conducted in accordance with the Declaration of Helsinki. Ethics Committee approval was obtained from the University Hospital of the Federal University of Maranhão—UFMA (CEP-HU/UFMA; Opinion No. 6834256, approved on 20 May 2024). Written informed consent for publication was obtained from the patient’s legal guardians.

## 3. Discussion

ICH is one of the most feared complications of cryptococcal meningoencephalitis, occurring in up to 56% of patients [11]. Its pathophysiology is multifactorial: the high fungal burden in the CSF impairs reabsorption at the arachnoid villi, raises CSF viscosity, and may directly obstruct CSF outflow pathways [1,3,14,15]. Additional contributors include interstitial cerebral edema, communicating hydrocephalus, and perivascular fungal infiltration [2,12]. In our case, we propose a possible clinical-manometric ICP dissociation—defined as clinical signs suggestive of raised ICP in the presence of consistently normal lumbar opening pressures. This dissociation, however, remains inferred and is explicitly not proven, as no reference invasive ICP measurement was obtained. Critically, gadolinium enhancement of the optic nerve sheath—as observed on MRI in our patient—reflects perineural inflammation or leptomeningeal infiltration rather than elevated intracranial pressure per se; optic nerve sheath distension, not enhancement, is the MRI correlate of raised ICP. Similarly, papilledema in the context of active cryptococcal leptomeningitis may be infiltrative in origin rather than pressure-driven, particularly in the absence of posterior globe flattening [4]. These considerations mean that the imaging and fundoscopic findings, while consistent with a severe inflammatory process, do not independently confirm true ICP elevation, and further qualify the proposed dissociation as a hypothesis.

Two main mechanisms may account for the observed possible dissociation between clinical signs suggestive of ICH and lumbar opening pressures. Adhesive arachnoiditis—causing spinal CSF compartmentalization that uncouples lumbar from intracranial pressure—is arguably the most biologically plausible mechanism, given the propensity of cryptococcal meningoencephalitis to produce basal and spinal fibrotic obstruction [4,11,14,16,17]. Pulsatile ICP dynamics and sampling bias during individual measurements represent a second, contributing mechanism. Neither can be distinguished from direct perineural and encephalitic disease without simultaneous invasive lumbar and intracranial pressure measurements, and both remain hypothetical in the absence of such data. In addition, the opening pressures recorded after day four were obtained with the drain clamped for at least six hours prior to measurement; this condition may not fully reflect the patient’s untreated intracranial dynamics and represents a further limitation on the interpretation of those values [18].

The species-level attribution to *C. gattii* carries important clinical implications. Unlike *C. neoformans*, which overwhelmingly affects immunocompromised hosts, *C. gattii* is a true primary pathogen capable of causing invasive CNS disease in immunocompetent individuals. In the pediatric literature, cases of cryptococcal meningitis have been reported with *C. neoformans*, *C. gattii*, *C. laurentii*, *C. albidus* and *C. humicola*, underscoring the importance of species identification in pediatric cases [3,10]. *C. gattii* is associated with greater neurotropism, a stronger inflammatory host response, and a high propensity for perineural leptomeningeal dissemination affecting the optic, auditory, and other cranial nerves in up to 40% of cases [2,13,19,20], directly responsible for the irreversible amaurosis and bilateral sensorineural hearing loss observed in our patient. The most common neurological sequelae in pediatric survivors of cryptococcal meningitis include visual impairment (highly correlated with raised ICP), sensorineural hearing loss, learning difficulties, and recurrent seizures [3,10,21,22]. Systematic audiological and neuro-ophthalmological follow-up, combined with early rehabilitation referral, is therefore mandatory in all pediatric cases. Anti-GM-CSF autoantibody testing excluded a cryptic immune defect predisposing to *C. gattii* infection [14,23].

Regarding antifungal therapy, the 2022 WHO guideline—which recommends the single 10 mg/kg dose of liposomal Amphotericin B from the AMBITION-CM trial—specifically applies to adults, adolescents, and children living with HIV; its application to non-HIV cryptococcal meningitis is not supported by current evidence [24,25]. The 2024 ECMM/ISHAM/ASM Global Guideline for cryptococcosis explicitly states that the AMBITION-CM regimen should not be extrapolated to non-HIV patients, and recommends liposomal Amphotericin B 3–4 mg/kg/day plus Flucytosine for the induction phase in non-HIV CNS cryptococcosis, with prolonged induction particularly considered for *C. gattii* disease [12]. In our patient, the escalated regimen (liposomal Amphotericin B 3 mg/kg/day plus Flucytosine) was therefore consistent with the most applicable current guideline.

ICP management follows a stepwise approach: serial therapeutic lumbar punctures target an opening pressure below 20 cm H_2_O or a ≥50% reduction when markedly elevated [11]. When this strategy fails, the current recommendation is escalation to continuous lumbar subarachnoid drainage, and when drainage fails or is complicated, to ventriculoperitoneal (VP) shunting [2,11,26]. In our patient, the external lumbar subarachnoid drain provided only transient benefit and was complicated by drain-associated meningitis caused by *P. aeruginosa*. VP shunting was discussed but not performed in the acute phase because of the concurrent active CNS infection and the absence of progressive ventriculomegaly on imaging; it remains an option for future management if obstructive hydrocephalus develops. Invasive ICP monitoring was not pursued due to concern about seeding an active CNS infection and limited local availability. These decisions represent real limitations of the management of this case and are highlighted in the limitations section. A landmark series by O’Brien et al. reported three pediatric *C. gattii* cases with persistent raised ICP, two of whom required VP shunting and all of whom developed IRIS [26].

The inadequacy of intermittent manometry for longitudinal ICP guidance provides a compelling argument for continuous noninvasive ICP monitoring. Ballestero et al. [18] demonstrated that noninvasive waveform morphology analysis reliably predicts impending ICH episodes in children with hydrocephalus. The brain4care sensor validated by Brasil et al. [27] reconstructs a real-time ICP waveform including the P2/P1 amplitude ratio—a validated index of reduced intracranial compliance that rises before absolute pressure thresholds are breached. It must be acknowledged, however, that the proposed dissociation in our case remains inferred rather than directly demonstrated, as no validated reference ICP measurement (invasive or noninvasive) was available for comparison. The utility of continuous noninvasive monitoring in active CNS infection with CSF compartmentalization is also unproven and represents a hypothesis rather than an established recommendation.

The diagnosis of suspected paradoxical IRIS in *C. gattii* meningoencephalitis requires careful distinction from treatment failure and disease progression due to perineural dissemination. IRIS criteria—paradoxical worsening with improving microbiological markers and an intensified inflammatory CSF profile—were met in our patient [26,28,29]. However, the absence of meaningful clinical response to pulse methylprednisolone and IVIG, combined with MRI-confirmed progressive perineural enhancement, suggests that structural cranial nerve injury from leptomeningeal dissemination was the predominant driver. Importantly, the IRIS diagnosis in this case must be qualified as “suspected” given the absence of a validated diagnostic biomarker and the plausibility of overlapping inflammatory and structural mechanisms [11,26]. This case illustrates the diagnostic complexity of distinguishing true IRIS from disease progression and argues for a stringent diagnostic framework—excluding treatment failure, antifungal resistance, and secondary infections—before initiating immunosuppressive therapy.

## 4. Limitations

Several limitations of this report must be acknowledged. First, this is a single-patient case report; generalization is inherently limited. Second, the proposed clinical-manometric dissociation remains inferred: no reference invasive or validated noninvasive ICP measurement was obtained to confirm true ICP elevation; direct perineural and encephalitic disease cannot be excluded as predominant contributors. Third, manometry conditions were not fully standardized across all measurements; opening pressures after day four were obtained with the drain clamped for ≥6 h, which may not reflect the untreated intracranial pressure state. Fourth, gadolinium enhancement of the optic nerve sheath is not a specific marker of raised ICP and may instead indicate perineural inflammation or infiltration; similarly, papilledema in active leptomeningitis may be infiltrative rather than pressure-driven. These findings therefore do not independently confirm true ICH. Fifth, serum CrAg was not available at admission. Sixth, transorbital ONSD measurement was not performed. Seventh, actual MRI and India ink images could not be included due to institutional data governance constraints. Eighth, the clinical course was reconstructed retrospectively.

## 5. Conclusions

Pediatric cryptococcal meningoencephalitis caused by *C. gattii* is rare but carries a high burden of irreversible neurological sequelae in immunocompetent hosts [3,4,10,19,20]. This case illustrates a possible clinical-manometric ICP dissociation that, if confirmed in future cases, may reflect adhesive arachnoiditis with spinal CSF compartmentalization—a mechanism distinct from pulsatile ICP sampling bias and one with direct therapeutic implications. It must be acknowledged that this dissociation remains a hypothesis: gadolinium enhancement of the optic nerve sheath and papilledema in this clinical context may both reflect leptomeningeal infiltration rather than raised ICP. Clinicians should be aware that normal lumbar opening pressures do not exclude clinically significant ICH in this disease. Antifungal therapy should be guided by the 2024 ECMM/ISHAM/ASM Global Guideline for non-HIV CNS cryptococcosis; CSF diversion should be considered early; and suspected paradoxical IRIS should be diagnosed cautiously, with rigorous exclusion of structural disease progression. Systematic audiological and neuro-ophthalmological follow-up with early rehabilitation is mandatory in all pediatric survivors [3,10]. Prospective studies on ICP monitoring strategies, including validated noninvasive approaches [18,27], are urgently needed.

## Figures and Tables

**Figure 1:**
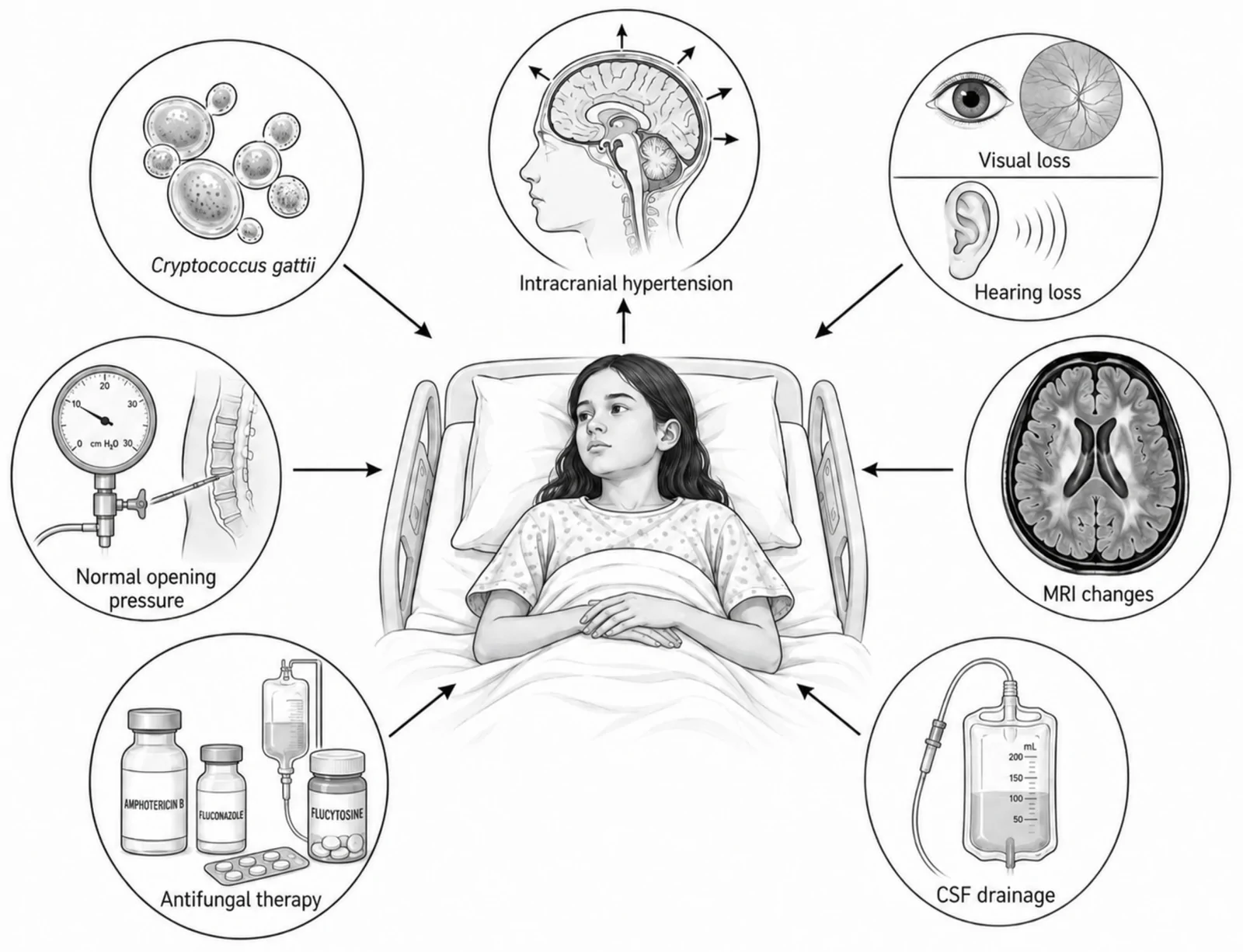
Schematic overview of the clinical presentation and management of pediatric cryptococcal meningoencephalitis caused by *Cryptococcus gattii*. Important note: This is a schematic AI-generated diagram (ChatGPT-4o, OpenAI, 2024) intended solely as a conceptual visual aid. It does not represent actual clinical images from this patient and should not be interpreted as original radiological or microbiological evidence. The MRI findings and India ink microscopy results described in the Case Report are based on the patient’s clinical records and are not depicted photographically in this report due to institutional data governance constraints. The central panel depicts the patient in the hospital setting surrounded by key elements: (i) encapsulated yeast (India ink, schematic); (ii) clinical signs suggestive of ICH despite normal manometric pressures; (iii) amaurosis and bilateral SNHL from perineural leptomeningeal dissemination; (iv) the possible clinical-manometric dissociation; (v) MRI enhancement of the right optic nerve sheath and cranial nerves VII/VIII (schematic representation); (vi) external lumbar subarachnoid drain; and (vii) antifungal therapy per 2024 ECMM/ISHAM/ASM guideline. Arrows pointing toward the central panel indicate the clinical, diagnostic, and therapeutic elements converging on the patient’s course; the outward-pointing arrows around the head (upper panel) represent raised intracranial pressure.

**Table 1:** Clinical timeline summary (CARE reporting format). GCS: Glasgow Coma Scale; OP: opening pressure; CSF: cerebrospinal fluid; LP: lumbar puncture; AmBd: Amphotericin B deoxycholate; L-AmB: liposomal Amphotericin B; 5-FC: Flucytosine; FLC: Fluconazole; #, sequential number of the lumbar puncture.

Day	Symptoms/GCS	OP (cm H_2_O)	CSF (Cells/μL; Glucose; Protein)	India Ink/Culture	Antifungal Therapy	Key Events
1	Headache, vomiting, asthenia; GCS 14	16	464; 31 mg/dL; 62.8 mg/dL	India ink: yeast/Pending	Ceftriaxone (empirical)	Admission; LP #1
2	Worsening headache; GCS 13	18	—	—	AmBd + FLC initiated	LP #2; papilledema on fundoscopy
3	Progressive headache, vomiting; GCS 12	17	—	—	AmBd + FLC	LP #3
4	Decreased consciousness; GCS 10	19	—	Culture: *C. gattii*	AmBd + FLC	ICU admission; intubation; external lumbar subarachnoid drain placed (L3–L4)
5–25	Mechanically ventilated; GCS 8–10	<20 (all)	—	Culture positive → negative wk 6	Escalated to L-AmB + 5-FC (wk 2)	Drain maintained; papilledema persists
21	—	—	—	—	L-AmB + 5-FC	Drain replaced (drain-associated meningitis, *P. aeruginosa*); Meropenem started
~Day 28	Progressive visual + auditory loss	—	—	—	L-AmB + 5-FC	MRI: hydrocephalus, perineural enhancement (CN II, VII, VIII), dilated perivascular spaces
Wk 6	Stable; no fever	—	Yeast on India ink; culture –	—	Pulse methylprednisolone + IVIg (IRIS suspected, no response)	Immunological evaluation: no immunodeficiency; anti-GM-CSF
Wk 8+	Improving consciousness	—	—	—	FLC (maintenance) → L-AmB 3×/wk (hepatotoxicity)	Switched due to grade 3 ALT elevation
8 mo FU	Bilateral amaurosis; bilateral SNHL	—	—	—	—	Audiometry: severe-to-profound SNHL bilaterally; referral for cochlear implant and low-vision rehab

## Data Availability

Data are contained within the article.

## Abbreviations

The following abbreviations are used in this manuscript:

Anti-GM-CSF	Anti-granulocyte-macrophage colony-stimulating factor
CARE	Case Report guidelines (CAse REport)
CrAg	Cryptococcal antigen
CSF	Cerebrospinal fluid
ICH	Intracranial hypertension
ICP	Intracranial pressure
ICU	Intensive care unit
IRIS	Immune reconstitution inflammatory syndrome
MRI	Magnetic resonance imaging
ONSD	Optic nerve sheath diameter
SNHL	Sensorineural hearing loss
ULN	Upper limit of normal
VP	Ventriculoperitoneal
WHO	World Health Organization
